# Transcriptomic and metabolomic insights on the molecular mechanisms of flower buds in responses to cold stress in two *Camellia oleifera* cultivars

**DOI:** 10.3389/fpls.2023.1126660

**Published:** 2023-02-20

**Authors:** Ya-Jun Wang, Ling-Li Wu, Min-hong Sun, Ze Li, Xiao-Feng Tan, Jian-An Li

**Affiliations:** ^1^ Key Laboratory of Cultivation and Protection for Non-wood Forest Trees, Ministry of Education, and the Key Laboratory of Non-Wood Forest Products, Forestry Ministry, Central South University of Forestry and Technology, Changsha, China; ^2^ Engineering Technology Research Center of Southern Hilly and Mountainous Ecological Non-Wood Forest Industry of Hunan Province, Changsha, China; ^3^ Camellia Oil Tree Research Institute of Central South University of Forestry and Technology, Changsha, China; ^4^ The Belt and Road International Union Research Center for Tropical Arid Non-wood Forest in Hunan Province, Changsha, China

**Keywords:** *Camellia oleifera*, cold stress, phenylpropanoid, plant hormones, starch and sucrose, metabolome, transcriptome

## Abstract

**Introduction:**

The *Camellia oleifera* (*C. oleifera*) cultivars 'Huashuo' (HS) and 'Huaxin' (HX) are new high-yielding and economically valuable cultivars that frequently encounter prolonged cold weather during the flowering period, resulting in decreased yields and quality. The flower buds of HS sometimes fail to open or open incompletely under cold stress, whereas the flower buds of HX exhibit delayed opening but the flowers and fruits rarely drop.

**Methods:**

In this study, flower buds at the same development stage of two *C. oleifera* cultivars were used as test materials for a combination of physiological, transcriptomic and metabolomic analyses, to unravel the different cold regulatory mechanisms between two cultivars of *C. oleifera*.

**Results and discussion:**

Key differentially expressed genes (DEGs) and differentially expressed metabolites (DEMs) involved in sugar metabolism, phenylpropanoid biosynthesis, and hormone signal transduction were significantly higher in HX than in HS, which is consistent with phenotypic observations from a previous study. The results indicate that the flower buds of HX are less affected by long-term cold stress than those of HS, and that cold resistance in *C. oleifera* cultivars varies among tissues or organs.This study will provide a basis for molecular markers and molecular breeding of *C. oleifera*.

## 1 Introduction

*Camellia oleifera* Abel. (*C*. *oleifera*) is a member of the genus *Camellia* in the family Theaceae. *C. oleifera* is one of four major woody oil tree species in China and has a wide range of applications. The main product from *C*. *oleifera* is tea oil, which is extracted from the seeds. Tea oil is a high-quality edible oil containing a significant proportion of unsaturated fatty acids (>90%), primarily linoleic acid and oleic acid (>80%), as well as squalene, tea polyphenols, tocopherol, and phytosterol (Liu et al., 2018; Zhang et al., 2022). Tea oil induces antioxidant enzymes *in vitro* and *in vivo*, and protects against oxidative damage to liver tissues and gastrointestinal mucosa (Teixeira and Sousa, 2021). It is frequently referred to as ‘‘Oriental olive oil’’ due to its high oleic acid content. Numerous byproducts are produced in the process of extracting tea oil from the seeds, including tea meal and tea shell. The latter is an important industrial raw material for extracting tea saponin, which is widely used in the production of laundry products, organic fertilizers, and insecticides (Quan et al., 2022). *C. oleifera* is currently cultivated in red soils in hilly areas throughout much of southern China. Hunan, Jiangxi, and Guangxi provinces are the primary production areas and account for 76% of the total area of production in the country (Qin et al., 2018). Hunan has a subtropical monsoon humid climate and four distinct seasons, with mean temperatures of 10–12°C in autumn and winter and 4–8°C in January. *C. oleifera* typically flowers from October to February, and thus frequently encounters cold temperatures during the flowering period (Gao et al., 2015).

Cold is among the most important abiotic stresses affecting plants, and can negatively impact overall growth and development. Consequently, the growth of nutritional (leaves) and reproductive organs (flowers) can be inhibited when plants are exposed to temperatures below their optimal growth temperatures, resulting in reduced yields (Tuteja et al., 2011; Soualiou et al., 2022). Studies focused only on physiological responses to stress cannot identify the mechanisms by which plants adapt to environmental stresses, and reproductive traits may be a better marker of plant adaptive responses (Kumar et al., 2012). The reproductive stages of flowering plants are typically highly sensitive to temperature, and cold frequently leads to delayed flowering, induces pollen sterility, and disrupts mitosis I and II (Zinn et al., 2010). Furthermore, studies have shown that while dormant buds are not sensitive to cold temperatures, expanding buds and flowers are vulnerable to cold. For example, cold can delay the development of apple (*Malus domestica*) buds, and the flowers are more cold-sensitive during the active period than during the dormant period (Proebsting and Mills, 1978; Salazar-Gutiérrez et al., 2016).

**Figure 1:**
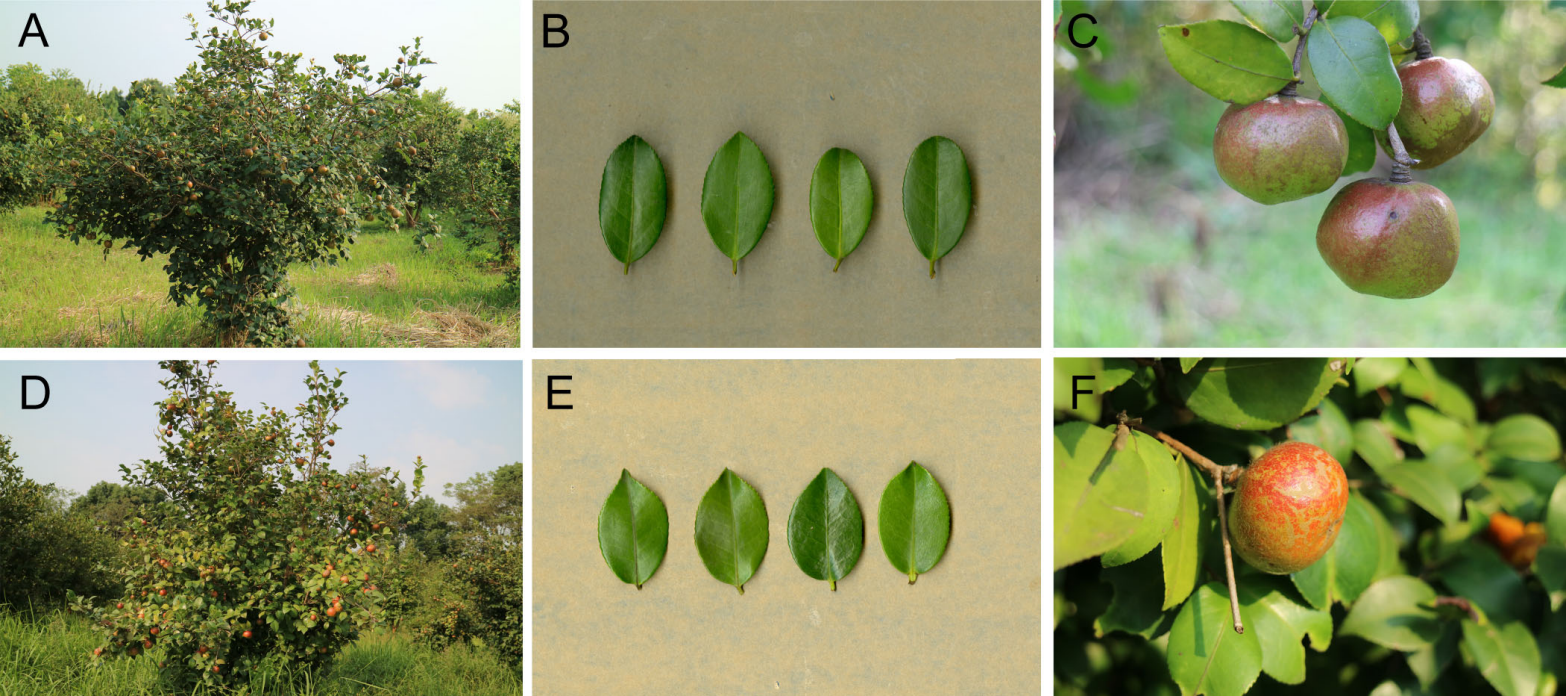
Trees, leaves, and fruits of HS **(A–C)** and HX **(D–F)**.

The *C*. *oleifera* cultivars ‘Huashuo’ (HS) and ‘Huaxin’ (HX) are new, high-yielding national cultivars bred from common *C*. *oleifera* seedling (Tan et al., 2011; Tan et al., 2012). HS forms a half open round crown, and has smooth, yellowish-brown bark (**Figure 1A**). The leaves are oval and dark green in color, with an average thickness of 0.48 mm (**Figure 1B**). This cultivar generally flowers from early November to early December and the fruit is a yellow-brown, five-sided ovate capsule (**Figure 1C**). Average fruit weight can reach 68.75 g at the height of the fruiting season. HX is tall tree with a naturally round crown and yellow-brown bark (**Figure 1D**). The leaves are leathery, broadly ovate, and dark green in color, with an average thickness of 0.44 mm (**Figure 1E**). The flowering period generally lasts from October to mid-December, and the fruit is ovate and green-yellow in color (**Figure 1F**). Average fruit weight can reach 48.83 g at the height of the fruiting season. These two cultivars are currently cultivated throughout Hunan, as 8–14°C is the optimum flowering temperature for *C*. *oleifera*, and damage due to cold stress can significantly affect its reproductive output.

Research into the cold resistance of *C*. *oleifera* cultivars has mainly focused on physiological and biochemical responses in vegetative growth organs (mainly leaves), whereas little research has been conducted on the molecular mechanisms involved in cold stress responses during the flowering period (Dong et al., 2020). Previous studies have shown that under cold stress, the flower buds of HS fail to open, open only partially, or wilt and drop. However, whereas the flowers HX exhibit delayed opening, the flowers and fruits of this cultivar exhibit little dropping due to the presence of a nectar-like sticky material at the base of the flowers, which may be a physiological response that protects the floral apparatus from cold damage (Wu et al., 2020a). In addition, biochemical reactions to cold stress differed between the two cultivars (Wu et al., 2020b).

To explaining the differences of phenotypes from the molecular perspective, we investigate changes in metabolism and transcriptional levels in the flower buds of the two cultivars under cold stress, we used flower buds as test materials, and performed untargeted metabolomics and transcriptomics using ultra-performance liquid chromatography-tandem mass spectrometry (UPLC-MS/MS) and Illumina next generation sequencing (NGS) technology. Our analyses will provide a basis for molecular markers and molecular breeding of *C*. *oleifera*.

## 2 Materials and methods

### 2.1 Plant materials and cold treatments

Samples of HX and HS were obtained from the Seedling Center of Hunan. In March 2018 we selected 120 robust, 2-year-old oil seedlings and transplanted them into plastic pots (22 × 22 × 20 cm) filled with a mix of peat, loess, and perlite (2:1:1). Transplants were placed on the roof of the Life Science Building at the Central South University of Forestry and Technology (Changsha, Hunan, 28° 10′ N, 113° 23′ E) and were subject to similar water and fertilization regimes. The cold stress experiment was conducted between November 2019 and January 2020 in an artificial climate chamber. On November 3, 2019, we selected 40 dwarfed but robust plants (20 plants per cultivar) with numerous flower buds, and exposed them to cold stress treatments (6°C) in an artificial climate chamber. Other parameters in the chamber were as follows: relative humidity = 70–80%, photoperiod = 12 h (8:00 a.m.–8:00 p.m.), photon flux density = 200 μmol·m^–2s^, and average carbon dioxide concentration = 450 μmol·mol^–1^. No fertilizer was applied during the cold treatments. Plants were given 500 mL water every 3 days to maintain soil moisture.

Samples were consistently collected in the same order and processed at approximately 10:00 a.m. Six samples of each cultivar were collected and each sample contained three unopened buds during four batch of sampling: the first was collected prior to placing plants into the artificial climate chamber as CK (0 days) with 6 samples labelled CK_HX1 to CK_HX6 and CK_HS1 to CK_HS6, the second batch was collected after 1 day in the artificial climate chamber as ST (1 days; short-term stress) with 6 samples labelled ST_HX1–ST_HX6 and ST_HS1–ST_HS6, the third after 7 days as MT (7 days; medium-term stress) with 6 samples labelled MT_HX1–MT_HX6 and MT_HS1–MT_HS6, and the fourth after 25 days as LT (long-term stress; LT) with 6 samples labelled LT_HX1–LT_HX6 and LT_HS1–LT_HS6. Samples were immediately wrapped in tin foil and labeled, snap-frozen in liquid nitrogen for 30 min, and then stored at –80°C in an ultra-cold freezer. Each sample was divided into two parts, one for untargeted metabolomics and the other for transcriptomics.

### 2.2 Sugar and endogenous hormone measurements

The 24 samples obtained from this experiment were analyzed by gas chromatography (GC)-MS for the absolute quantification of 13 sugars and UPLC-MS/MS for the absolute quantification of three plant hormones (**Supplementary Table S1**). Each test was replicated three times and followed methods described in a previous study (Wu et al., 2022).

### 2.3 Metabolomic sample preparation and extraction

The samples were freeze-dried and crushed for 1.5 min at 30 Hz using a mixer mill (MM 400; Retsch GmbH, Haan, Germany) with a zirconia bead. We weighed 100 mg powder and extracted it overnight at 4°C in 0.6 mL 70% aqueous methanol. Following centrifugation at 10,000 g for 10 min, the extracts were absorbed (CNWBOND Carbon-GCB SPE Cartridge, 250 mg, 3 mL; ANPEL, Shanghai, China) and filtered (SCAA-104, pore size 0.22 μm; ANPEL) in preparation for the UPLC-MS/MS analysis.

The sample extracts were analyzed on a UPLC-ESI-MS/MS system (UPLC-MS/MS: Shim-pack UFLC SHIMADZU CBM30A system; Shimadzu, Kyoto, Japan; GC-MS: 4500 quadrupole-linear ion trap [Q TRAP]; Applied Biosystems, Waltham, MA, USA). The analytical conditions for UPLC were as follows. The column was an Acquity UPLC HSS T3 C18 column (1.8 µm, 2.1 mm × 100 mm; Waters Corporation, Milford, MA, USA). The mobile phase consisted of solvent A (pure water with 0.04% acetic acid) and solvent B (acetonitrile with 0.04% acetic acid). Acetic acid and acetonitrile were purchased from Merck (Darmstadt, Germany). Sample measurements were performed along a gradient, with the starting conditions 95% A and 5% B. Within 10 min, a linear gradient to 5% A + 95% B was applied, and the composition of 5% A + 95% B was maintained for 1 min. Then the composition was adjusted to 95% A + 5.0% B within 0.10 min and maintained for 2.9 min. The column oven was set to 40°C, and the injection volume was 4 µL. The effluent was alternatively connected to an ESI-triple Q TRAP-MS. The qualitative identification of metabolites is based on the database MWDB.

### 2.4 Library preparation and RNA sequencing

RNA samples were sent to Wuhan Metware Biotechnology Co., Ltd. (Wuhan, China), where the libraries were produced and sequenced. Total RNA was extracted from the flower buds using an RNA Prep Pure Plant Kit (Tiangen, Beijing, China) following the manufacturer’s protocols. RNA purity and concentration were determined using a NanoPhotometer spectrophotometer (Implen, Munich, Germany). The input material consisted of 3 µg RNA per sample. Sequencing libraries were generated using a NEBNext Ultra RNA Library Prep Kit for Illumina (New England Biolabs, Ipswich, MA, USA) following the manufacturer’s protocols. Library construction and inspection were conducted as previously described (Li et al., 2018). Polymerase chain reaction (PCR) products were purified using an AMPure XP system (Beckman Coulter, Brea, CA, USA) and library quality was assessed using an Agilent Bioanalyzer 2100 system (Agilent Technologies, Santa Clara, CA, USA). Libraries were sequenced on the Illumina HiSeq platform (Illumina Inc., San Diego, CA, USA) and 125 bp/150 bp paired-end reads were generated.

### 2.5 Transcriptome assembly and gene functional annotation

Clustering of the index-coded samples was performed using a cBot Cluster Generation System with a TruSeq PE Cluster Kit v3-cBot-HS (Illumina Inc.) following the manufacturer’s protocols. Following cluster generation, libraries were sequenced on the Illumina HiSeq platform and 125 bp/150 bp paired-end reads were generated. Image data obtained from the high-throughput sequencer were transformed into raw data using Casava base recognition. After filtering, the original data were used to determine the sequencing error rate and GC content distribution to obtain clean reads for subsequent analyses. Clean reads were assembled using a Trinity assembler, and the obtained transcripts were used as reference sequences in subsequent analyses. The transcripts were clustered hierarchically using read numbers and the expression patterns of the aligned transcripts. The longest cluster sequence obtained from corset hierarchical clustering was used as a unigene in subsequent analyses. Gene function was annotated using the NR, Pfam, euKaryotic Orthologous Groups (KOG), COG, eggNOG, Swiss-Prot, KEGG, and GO databases.

### 2.6 Differential expression analyses

Fragments per kilobase of transcript per million fragments mapped was used as a measure of transcript or gene expression level. DEGs between the two biological conditions were obtained by inter-sample group analysis using DESeq2 (Love et al., 2014). Following differential analysis, the false discovery rate (FDR) was obtained by correcting p-values for multiple hypothesis testing using the Benjamini-Hochberg method. The screening conditions for DEGs were |log2Fold Change| ≥ 1 and FDR < 0.05.

A combination of fold change and variance influence on projection (VIP) values from the orthogonal projections to latent structures discriminant analysis (OPLS-DA) model was used to identify DEMs. Metabolites with fold changes ≥ 2 or ≤ 0.5 were selected. Metabolites with a difference ≥ 2 or ≤ 0.5 in the control (CK) and experimental groups (ST, MT, and LT) were considered significantly different. Metabolites with VIP ≥ 1 were also selected.

### 2.7 Statistical analyses

**Figure 2:**
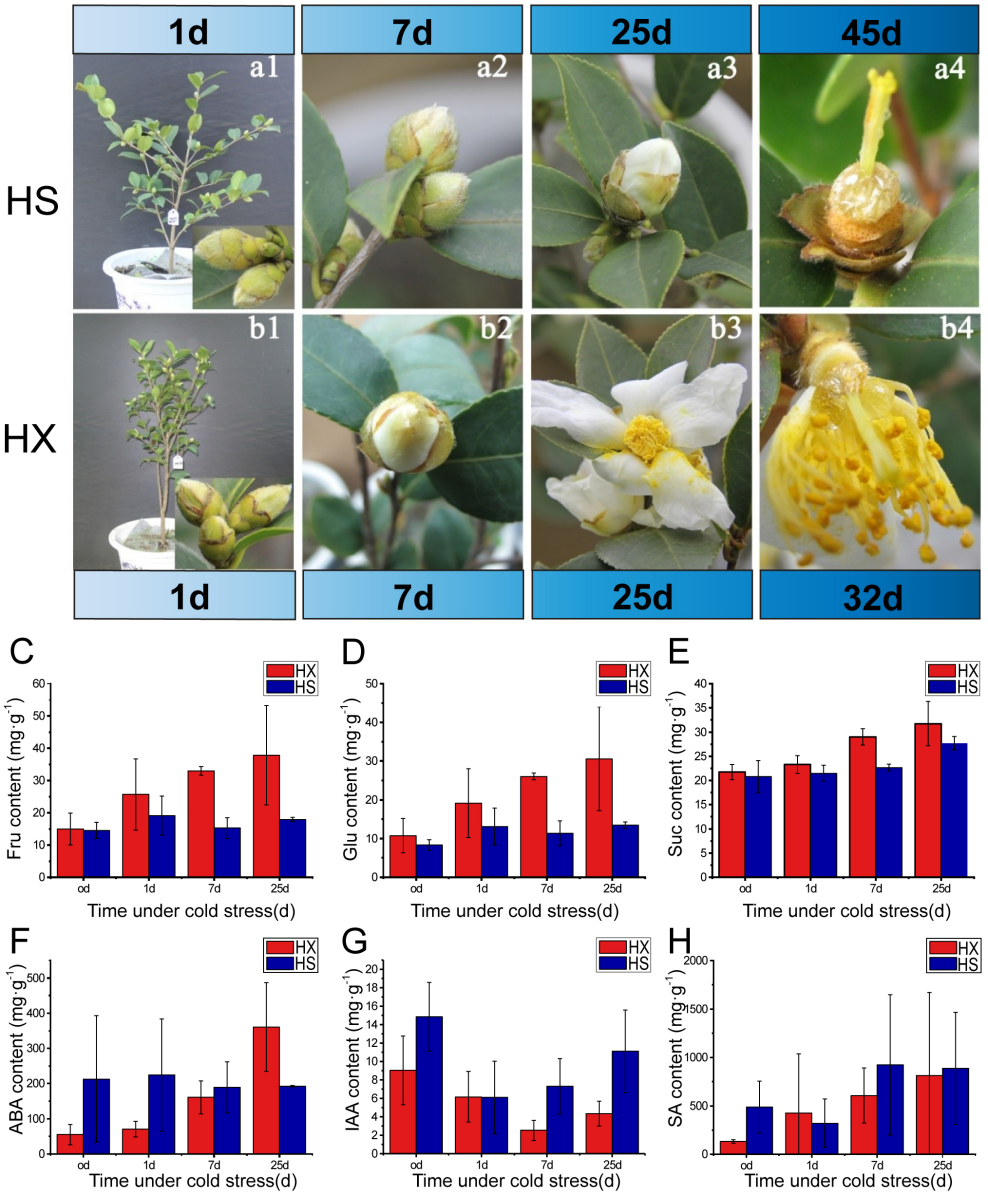
Effects of cold stress on phenotype, sugar content, and phytohormone content: bud morphology and flowering of HS **(a1-a4)**, and HX **(b1-b4)** during the treatment period; the contents of **(C)** D-fructose, **(D)** glucose, **(E)** sucrose, **(F)** ABA, **(G)** IAA, and **(H)** SA in flower buds of HS and HX under different cold treatments.

**Figure 3:**
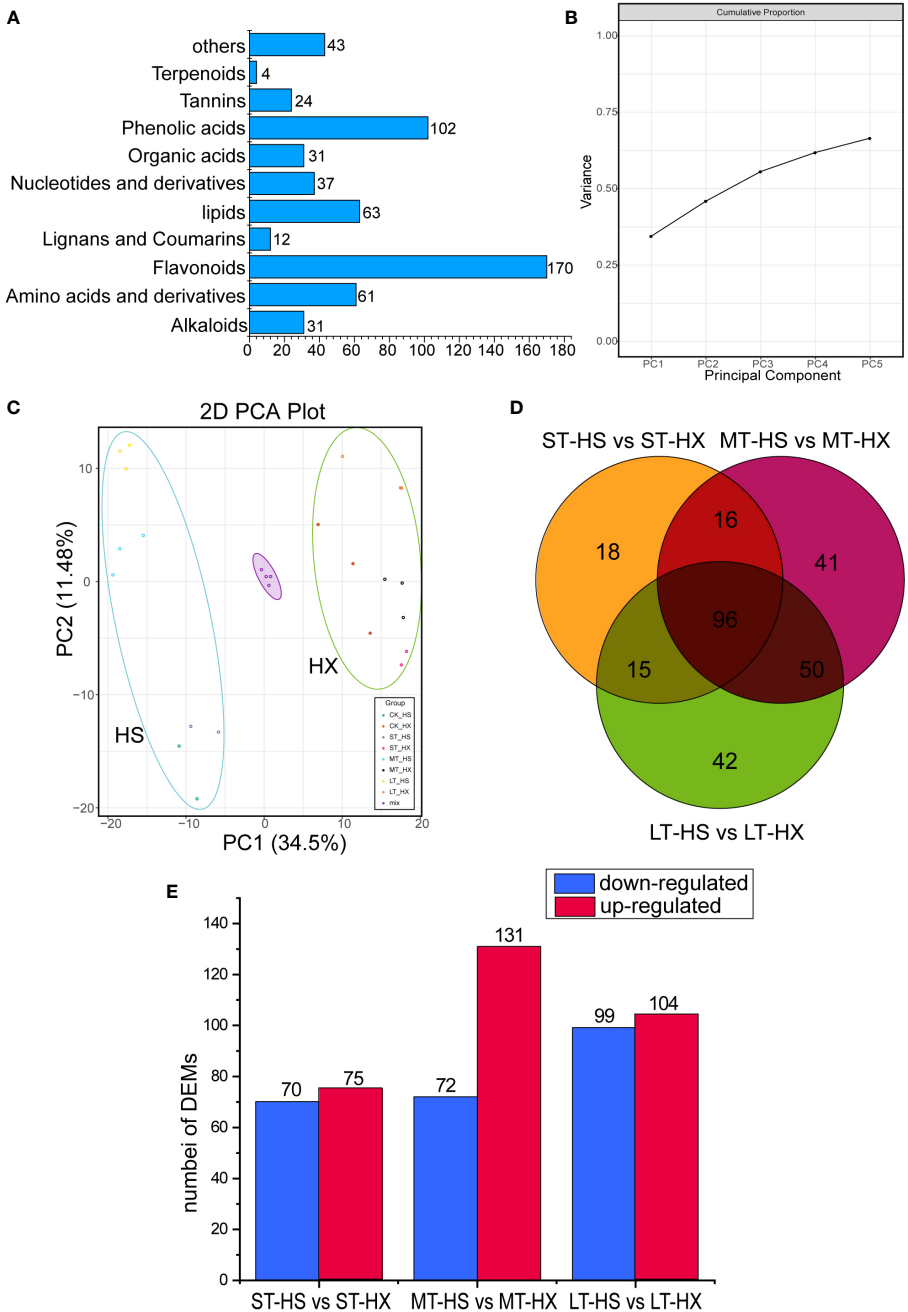
Overview and analysis of metabolites. **(A)** classification of the 578 metabolites. **(B)** variance explained by PCs 1–5. **(C)** PCA of metabolome data at the four sampling points (0, 1, 7, and 25 days; three biological replicates per point). **(D)** Venn diagrams of DEMs under different cold treatments. **(E)** up- and down regulated metabolites.

**Figure 4:**
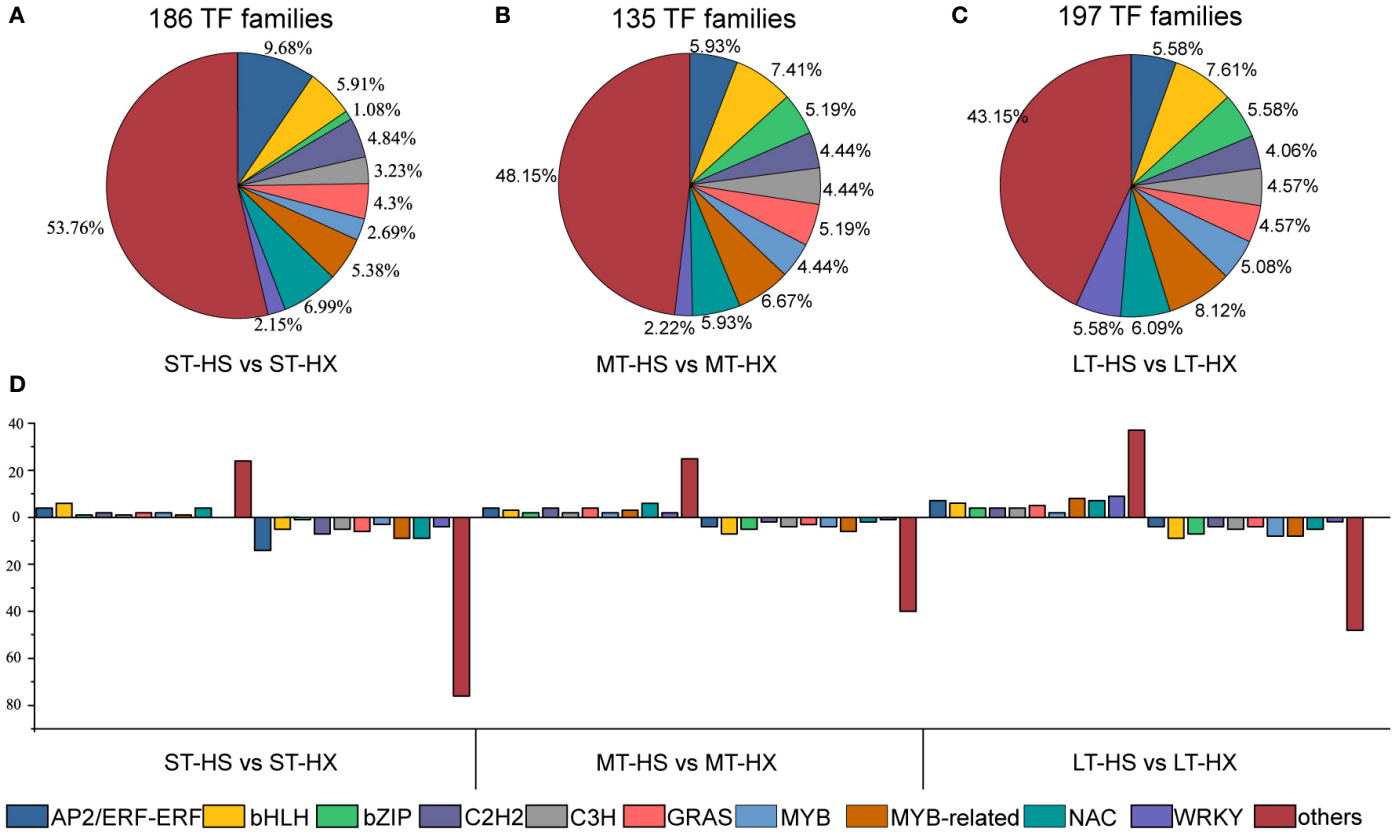
Cold stress-responsive TFs. **(A–C)** Distribution of TF families under ST, MT, and LT cold stress, and **(D)** up- and down regulated TF families.

**Figure 5:**
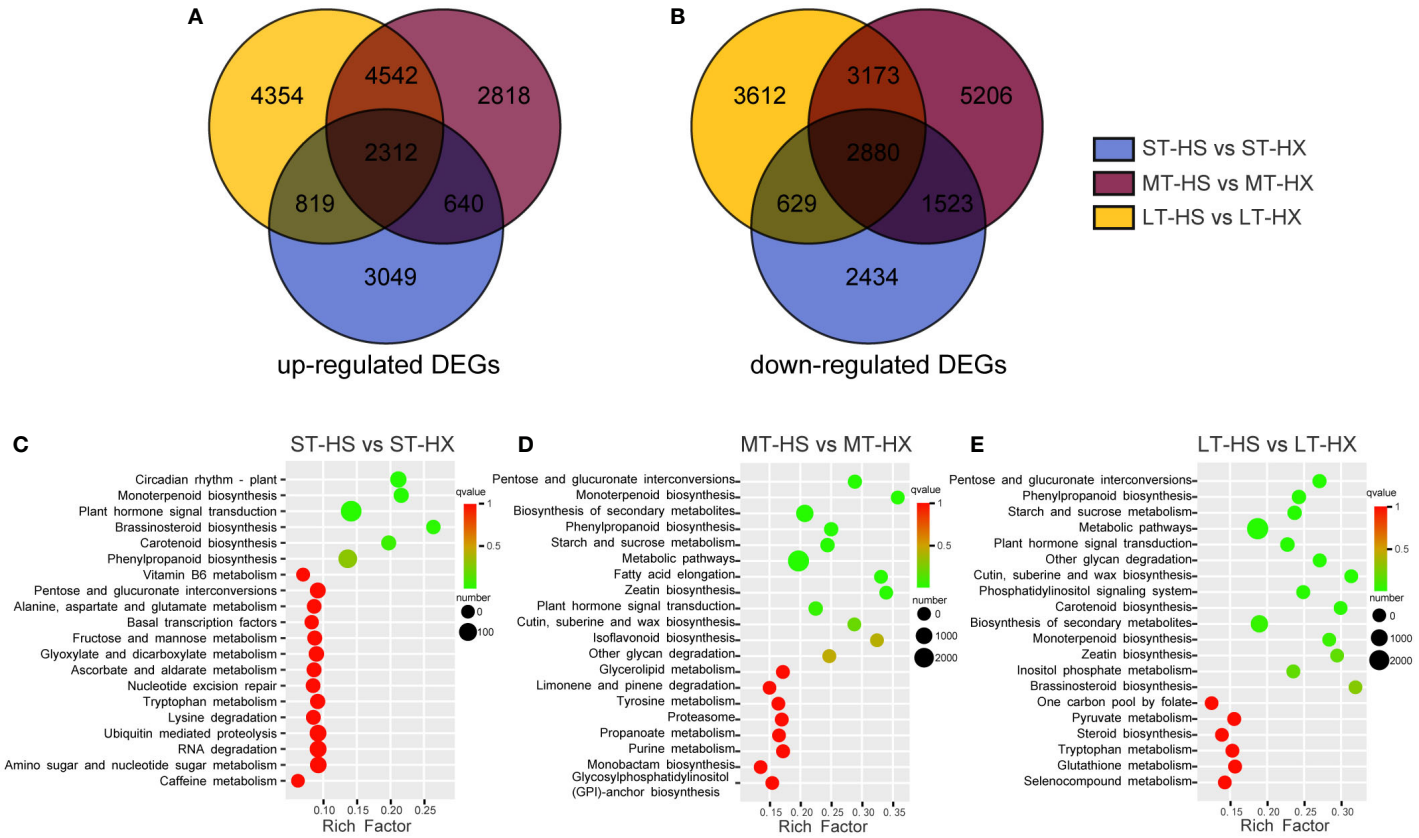
DEG analysis. The Venn diagram shows DEGs that were **(A)** up regulated or **(B) **down regulated; KEGG enrichment analysis of DEGs after **(C)** 1, **(D)** 7, and **(E)** 25 days of cold stress treatments.

**Figure 6:**
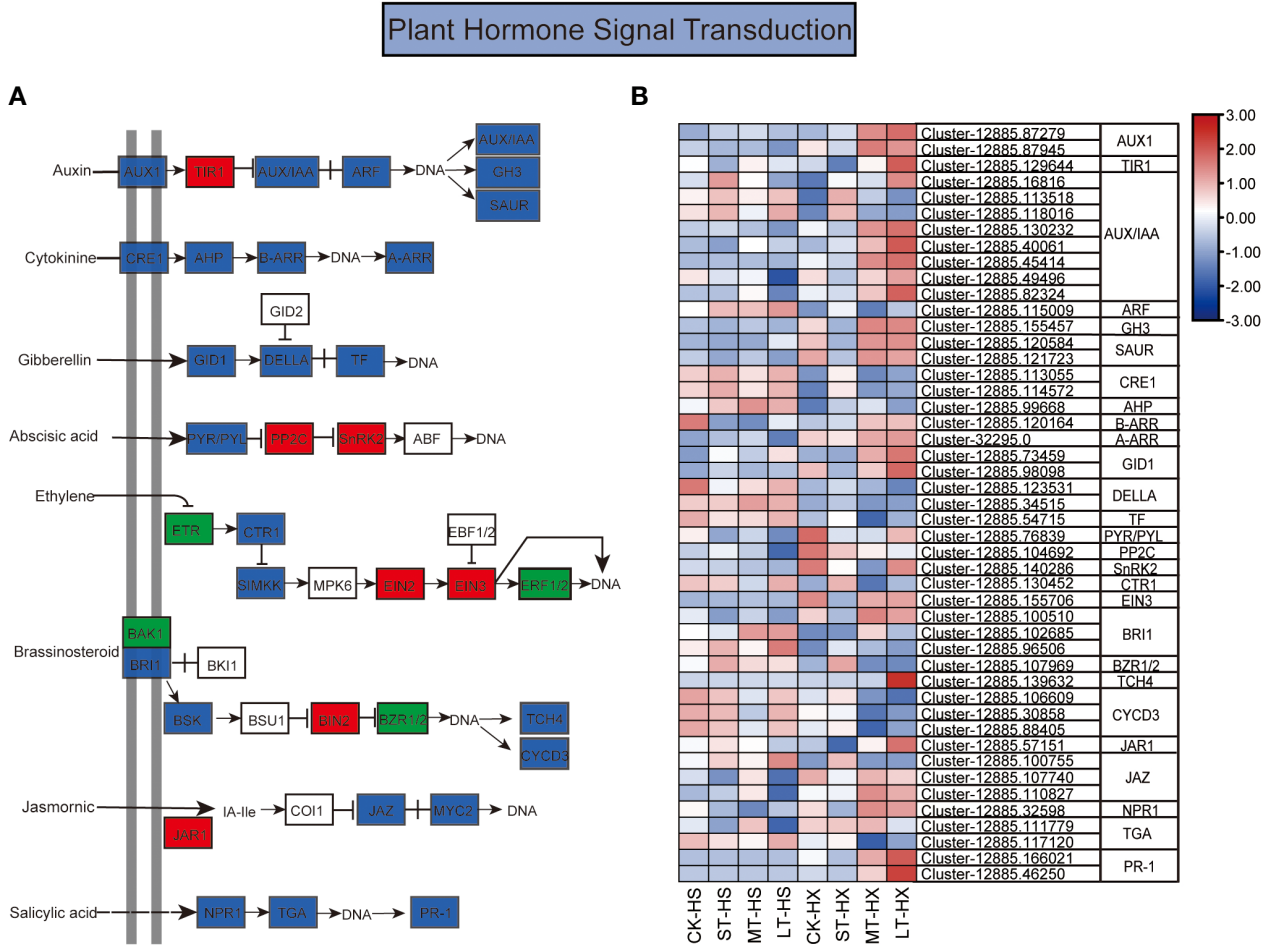
The KEGG pathway annotation diagram of the plant hormone signal transduction pathway of DEGs. **(A)** Schematic diagram of the KEGG pathway for the enrichment of DEGs in the plant hormone signal transduction pathway. Red boxes indicate upregulation, green boxes indicate downregulation, and blue boxes indicate both up- and downregulation. **(B)** Heatmap showing the expression of DEGs associated with different hormones. Box color indicates the expression level of each gene: blue = decreased expression and red = increased expression.

**Figure 7:**
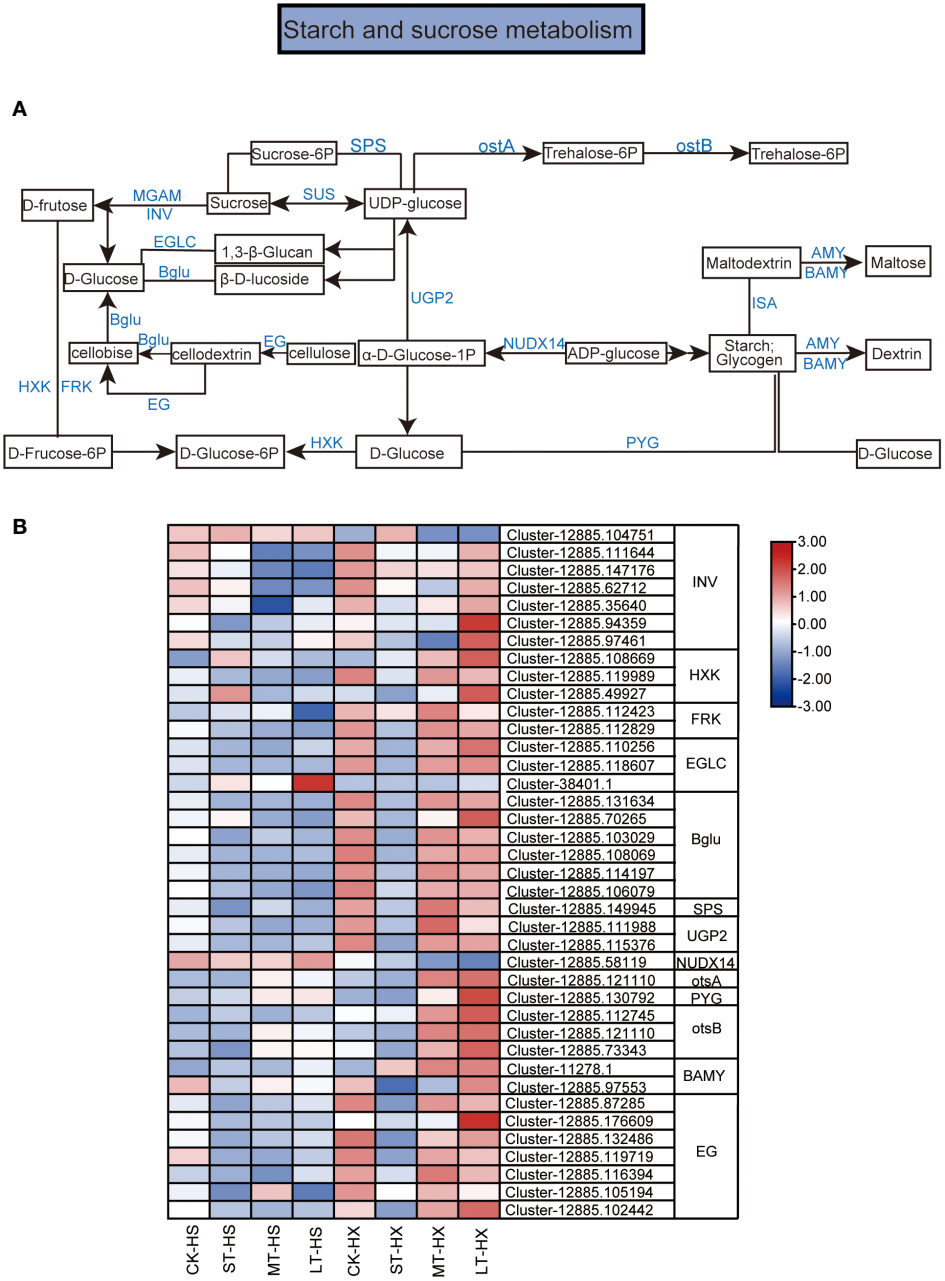
KEGG pathway annotation diagram for the starch and sucrose metabolism pathway of DEGs. **(A)** Schematic diagram of the KEGG pathway for the enrichment of DEGs in the starch and sucrose metabolism pathway. Blue text indicates enzymes and black text indicates metabolites. **(B)** Heatmap showing the expression of DEGs. Box color indicates the expression level of each gene: blue = decreased expression and red = increased expression.

**Figure 8:**
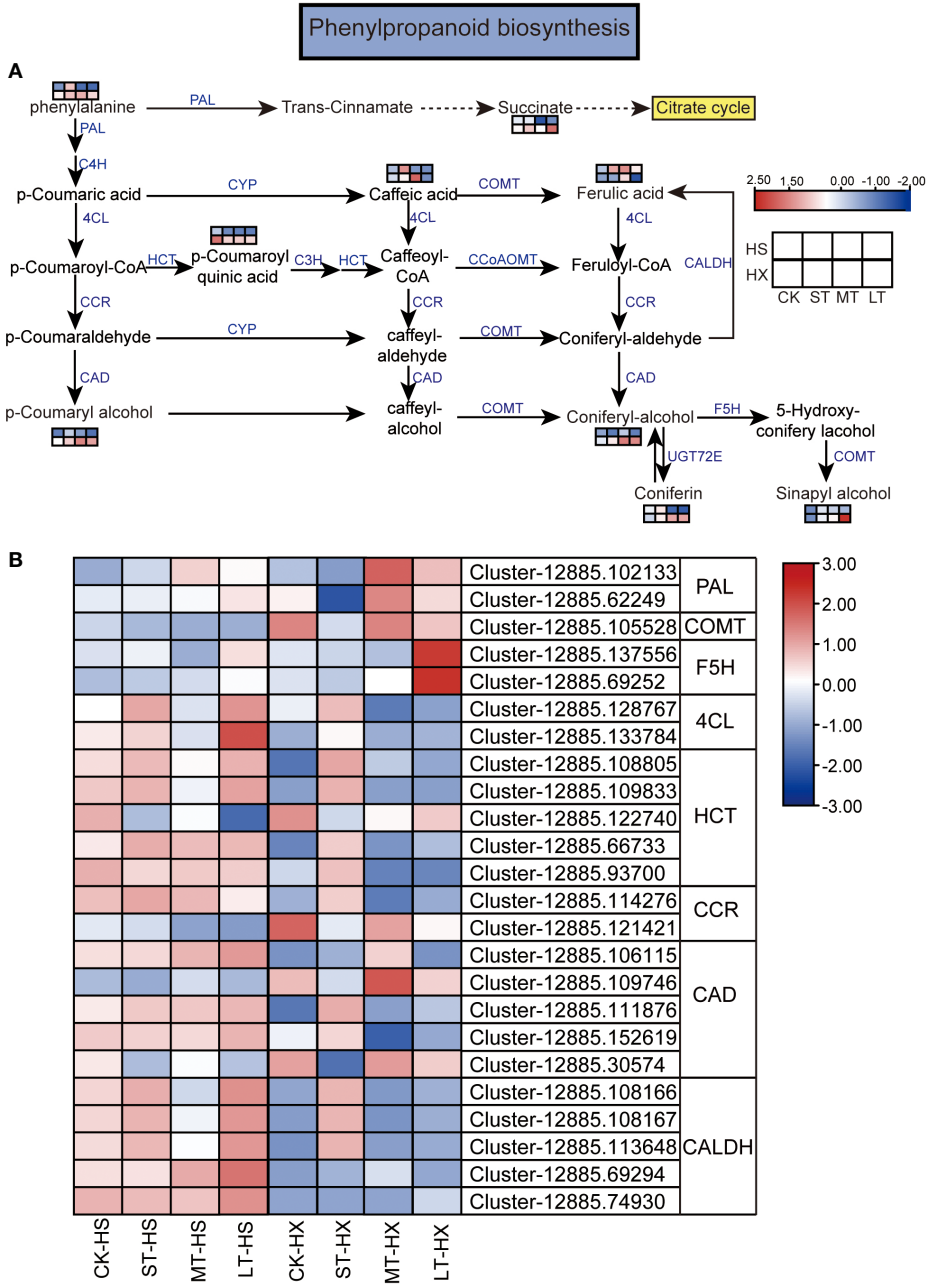
KEGG pathway annotation diagram of the phenylpropanoid biosynthesis pathway of DEGs and DEMs. **(A)** Schematic diagram of the KEGG pathway for the enrichment of DEGs and DEMs involved in phenylpropanoid biosynthesis. Blue text indicates enzymes, black text indicates metabolites, and the dotted lines indicate the omission of certain reactions. **(B)** Heatmap showing the expression of DEGs. Box color indicates the expression level of each gene: blue = decreased expression and red = increased expression.

Unsupervised principal component analysis (PCA) was conducted using the *prcomp* function in R (R: The R Project for Statistical Computing (r-project.org)). Set the prcomp function parameter scale=True, which means unit variance scaling (UV) normalization of data. The data were unit variance scaled prior to conducting the PCA. DEGs and DEMs were visualized in heat map form using TBtools v1.098763 software, and Venn diagrams were drawn using the Metware Cloud website (https://cloud.metware.cn/#/home). **Figures 1**, **2** were drawn in Adobe Photoshop 2020. **Figures 3**–**8** were drawn in Adobe Illustrator 2022.

## 3 Results

### 3.1 Phenotypic and physiological changes

The growth form of each cultivar prior to being placed in the artificial climate chambers is shown in **Figure 2**. The morphology and size of the flower buds were similar among the two cultivars. The calyx was wrapped around the buds and the calices and bracts were hairy. HS exhibited no obvious changes with respect the size of flower buds between Days 1 and 7 of the cold stress treatments, and the sepals remained closed and tightly wrapped around the internal flower structures (**Figures 2a1, a2**). However, a small number of sepals had unfolded by Day 25, exposing the petals (**Figure 2a3**). Some sepals gradually opened in HX, and the petals were visible in approximately one third of the buds by Day 7 (**Figure 2b2**). Most buds were completely open by Day 25 (**Figure 2b3**). The petals and stamens of HX dropped after 32 days of cold stress, and a honey-like mucilaginous substance appeared at the base of the flowers (**Figure 2b4**). This substance became stickier and more abundant as the duration of cold stress increased (observation period = 60 days). Observations indicated that the flower buds of HX opened one at a time under cold stress, whereas the flower buds of HS required an adaptive dormant period. Both cultivars bloomed under LT cold stress; however, the timing and duration of flowering differed.

To further assess changes in flower buds in response to cold stress, we measured the sugar and phytohormone contents of buds. Thirteen sugars and three phytohormones were detected. The three most abundant sugars were D-fructose, glucose, and sucrose, and most abundant phytohormones were salicylic acid (SA), abscisic acid (ABA), and indole-3-acetic acid (IAA). The contents of D-fructose, glucose, and sucrose in both cultivars increased after a single day of cold stress; however, while the contents of these sugars changed little in HS over the treatment period, they increased continuously in HX. Furthermore, contents of the three soluble sugars were consistently higher in HX than in HS, particularly after 25 days of treatment (**Figures 2C–E**). Cold stress promotes the accumulation of soluble sugars in HX, which may influence the cold tolerance of this cultivar. Phytohormones also differed between the two cultivars during cold stress: while the ABA content in HS did not vary significantly throughout the treatment period, it increased substantially in HX, peaking at 25 days and exceeding the levels observed in HS (**Figure 2F**). The trends in IAA content were similar among cultivars, first decreasing and then increasing; however, the IAA content of HS was higher than that of HX (**Figure 2G**). The SA content in HS first decreased and then increased as the duration of cold stress increased, but changes were not significant after 7 days, whereas the SA content in HX increased gradually with the lengthening of the duration of cold stress. SA content was higher in HS than in HX after 25 days of cold stress, but the difference was not significant (**Figure 2H**). These results indicate that differences in cold tolerance among the two cultivars might be related to sugar, ABA, and IAA.

### 3.2 Metabolite profile analysis

#### 3.2.1 Metabolite analysis

A total of 578 metabolites were detected. These metabolites were divided into 10 main groups, including alkaloids (31 metabolites), amino acids and derivatives (61), flavonoids (170), lignans and coumarins (12), lipids (63), nucleotides and derivatives (37), organic acids (31), phenolic acids (102), tannins (24), and terpenoids (4), plus an additional 43 compounds that did not fit into any of these groups (**Figure 3A**). The most abundant metabolites were flavonoids, phenolic acids, lipids, and amino acids and derivatives. PCA indicated that principal components 1–5 explained more than 62.5% of the cumulative variance in metabolites (**Figure 3B**). The PCA score plot indicated that the close pooling of the mixed group samples used for quality control demonstrated remarkable experimental repeatability; furthermore, principal components 1 and 2 accounted for 34.5% and 11.4% of variance, respectively. PC1 separated HS from HX, indicating that major differences in metabolite levels between the two cultivars, whereas PC2 separated samples exposed to cold stress for 0–1 day from those subjected to cold stress for 7–25 days (**Figure 3C**). In addition, while samples of both cultivars migrated upward along PC2 over the course of the treatment period, HS migrated farther than HX. Samples from the LT-HS group became increasingly clustered in the upper left as the duration of cold stress increased, suggesting that metabolic changes were more pronounced in HX than in HS.

We used a comparative analysis of changes in metabolites to identify differences in accumulation patterns between the cultivars in response to cold stress. We compared the DEMs of the two cultivars based on the criteria of fold change ≥ 2 or ≤ 0.5. We identified 145 DEMs after 1 day of cold treatment (75 upregulated and 70 downregulated), 203 DEMs after 7 days (131 upregulated and 72 downregulated), and 203 DEMs after 25 days (104 upregulated and 99 downregulated) (**Figure 3E**). Furthermore, 96 commonly enriched DEMs were identified in different comparisons, and 42 metabolites were identified specifically in the comparison of the LT-HS and LT-HX groups (**Figure 3D**). Most metabolites, including flavonoids, phenolic acids, and lipids, were more highly induced in HX than in HS, whereas the opposite was generally true for amino acids and derivatives. Moreover, the content of pyridoine, D-glucoronic acid, sodium ferulate, (R)-pantetheine, 5,7-dyhydroxy-1(3H)-isobenzofuran-one-O-glucoside, androsin, and D-(+)-melezitose were higher in HX than in HS, whereas nicotinic acid, riboflavin, and maltotetraose were more strongly induced in HS. Cold stress significantly induced certain metabolites in both cultivars, including L-Proline, succinic acid, and coniferin (**Supplementary Figure S1**).

#### 3.2.2 KEGG pathway mapping of DEMs

The KEGG pathways in different pairwise comparisons (ST-HS *vs* ST-HX, MT-HS *vs* MT-HX, and LT-HS *vs* LT-HX) typically included the valine, leucine, and isoleucine degradation, tyrosine metabolism, phenylpropanoid biosynthesis, and carbapenem biosynthesis pathways. The key enrichment pathways between ST-HS and ST-HX included the flavone and flavonol biosynthesis, anthocyanin biosynthesis, and ubiquinone and another terpenoid–quinone biosynthesis pathways, whereas the key enrichment pathways between MT-HS and MT-HX included the valine, leucine, and isoleucine degradation, phenylpropanoid biosynthesis, and valine, leucine and isoleucine biosynthesis pathways. Significantly enhanced pathways between LT-HS and LT-HX included the phenylpropanoid biosynthesis, propanoate metabolism, purine metabolism, and pyrimidine metabolism pathways (**Supplementary Figure S2**). The results indicate that metabolites in the flower buds of the two cultivars differ significantly under long-term cold stress.

### 3.3 Transcriptome analysis

#### 3.3.1 Transcription factors in response to cold stress

Transcription factors (TFs) are essential for gene expression in plants under abiotic stress, and a better understanding of TFs and their downstream target genes will facilitate the development of stress-tolerant crops with improved quality and yields (Khan et al., 2018). We identified 74 TF families in our analysis, of which the AP2/ERF-ERF family was the largest. Moreover, 186 TFs were identified in comparisons between the ST-HS and ST-HX groups (47 upregulated and 139 downregulated), 135 between the MT-HS and MT-HX groups (57 upregulated and 78 downregulated), and 197 between the LT-HS and LT-HX groups (93 upregulated and 104 downregulated) (**Figure 4D**). TF families that were susceptible to cold stress included MYB, MYB-related, GRAS, bHLH, C2H2, C3H, NAC, bZIP, WRKY, and AP2/ERF-ERF. In the comparison of ST-HS and ST-HX, the AP2/ERF-ERF family was the largest group (9.68%), followed by NAC (6.99%) and bHLH (5.91%) (**Figure 4A**). The four most abundant families in the MT-HS *vs*. MT-HX comparison were bHLH (7.41%), MYB-related (6.67%), AP2/ERF-ERF (5.93%), and NAC (6.09%) (**Figure 4B**). The three most abundant families in the LT-HS *vs*. LT-HX comparison were MYB-related (8.12%), bHLH (7.61%), and NAC (6.09%) (**Figure 4C**). Most genes in families such as AP2/ERF-ERF, MYB-related, NAC, and WRKY were more strongly upregulated in HX than in HS after 25 days of cold treatment, indicating that TF gene expression in the flower buds was more active in HX than in HS under long-term cold stress.

#### 3.3.2 DEGs analysis

The 24 samples were analyzed *via* transcriptome sequencing. After removing low-quality reads, a total of 1,393,597,534 clean data points were obtained, with the percentage of Q30 bases > 93% and the percentage of GC > 43.32%, indicating that the transcriptome sequencing was reliable and of high quality. Based on screening conditions |log2Fold Change| ≥ 1 and FDR < 0.05, 14,285 DEGs were identified between ST-HS and ST-HX (6,820 upregulated and 7,465 downregulated), 23,093 between MT-HS and MT-HX (10,312 upregulated and 12,781 downregulated), and 22,320 between LT-HS and LT-HX (22,320 upregulated and 22,320 downregulated) (**Supplementary Table S2**). In these three comparisons, 2,312 upregulated and 2,880 downregulated DEGs overlapped. Interestingly, 3,049, 2,818, and 4,354 DEGs were specifically upregulated after 1, 7, and 25 days of cold stress, respectively, whereas 2,434, 5,206 and 3,612 DEGs were specifically downregulated (**Figures 5A, B**). Upregulated genes became more numerous as the duration of stress increased, and may be among the key cold resistance mechanisms in HX. These results imply that the two cultivars respond differently to cold stress. The numerous DEGs shared among groups independent of the time of cold stress might be attributable to genetic differences between two cultivars. As such, the focus of our discussion from here will be the analysis of specific genes identified in the comparison of the LT-HS and LT-HX groups.

#### 3.3.3 Gene function annotation of DEGs

The unigene sequence was compared to the KEGG, NR, Swiss-Prot, GO, COG/KOG, and Trembl databases using BLAST software, and the amino acid sequence of the unigene was predicted and compared to the Pfam database using HMMER software to obtain annotation information for the unigene (**Supplementary Figure S3A**). The comparison of 10,951 transcripts with the NR library facilitated the identification of transcript sequence similarities between species and the obtention of functional information for homologous sequences. The results indicate that the species most similar to *C*. *oleifera* is *Vitis vinifera* (**Supplementary Figure S3B**). Following GO annotation of 234,040 unigenes, the annotated genes were classified based on the next level of three GO categories: biological processes, cellular components, and molecular functions. The terms with the highest number of transcripts in the biological processes category were “cellular and metabolic processes” and “biological regulation and stimulus-response” (**Supplementary Figure S3C**). For cellular components, the most abundant terms were “cell” and “cell portion,” whereas the most abundant term for molecular function was “binding.” A total of 52,336 transcripts were annotated and classified into 25 categories in the KOG database. Among these categories, general function prediction had the largest number of transcripts (11,774), followed by signal transduction mechanisms (5,078), posttranslational modification, protein turnover, and chaperones (4,909), and carbohydrate transport and metabolism (2,925) (**Supplementary Figure S3D**). These results indicate that sugar metabolism plays an important role in the responses of the two cultivars to cold stress. KEGG annotation-based enrichment analysis of the two cultivars at different points during the treatments revealed that DEGs were significantly enriched in the metabolic, secondary metabolic, starch and sucrose metabolism, plant hormone signal transduction, and phenylpropanoid biosynthesis pathways (**Figures 5C–E**). These results suggest that metabolites involved in starch and sucrose metabolism and plant hormone signal transduction may be key metabolites to the cold stress responses of the two cultivars.

#### 3.3.4 Plant hormone signal transduction pathways in response to cold stress

Hormones play a central role in regulating responses to cold stress in plants, which is essential to plant growth and development (Zhao et al., 2021). Our analysis identified a large number of DEGs between HS and HX under cold stress; these DEGs are involved in hormone signal transduction for a range of hormones, including IAA, ABA, cytokinin (CTK), gibberellin (GA), ethylene (ET),brassinosteroid (BR), jasmonic acid (JA), and SA (**Figure 6A**). The DEGs exhibited distinct expression patterns in the two cultivars, revealing complicated cold stress response mechanisms involving phytohormones. The expression of most DEGs was relatively stable in HS over the course of the treatments but exhibited significant upward trends in HX, peaking at 25 days. Fifteen DEGs in the auxin signal transduction pathway were significantly differentially expressed under different cold treatments, including genes encoding auxin influx carrier (AUX1), transport inhibitor response 1 (TIR1), auxin-induced protein/auxin-responsive protein (AUX/IAA), auxin response factor (ARF), auxin-responsive GH3 family (GH3), and SAUR family proteins (SAUR). The expression levels of two *AUX1* genes, one *TIR1* gene, six *AUX/IAA* genes, one *GH3* gene, and two *SAUR* genes were significantly higher in LT-HX than in LT-HS. By contrast, only two *AUX/IAA* genes and one *ARF* gene were downregulated in LT-HS compared to LT-HX. These results suggest that the expression of auxin signal transduction related genes was generally repressed in HS under prolonged cold stress. In the ABA pathway, DEGs including pyrabaction resistant/PYRlike (*PYR/PRL*), protein phosphatase 2C gene (*PP2C*), and sucrose non-fermenting 1-related protein kinase 2 gene (*SnRK2*) exhibited decreased expression in both HS and HX after 1 day of cold stress. Expression of these DEGs increased in HX throughout the treatment period, whereas in HS their expressions briefly increased and then decreased. Meanwhile, there was a significant increase in expression after 25 days in LT-HX compared to LT-HS. Other hormone signalling genes, including *B-ARR*, *A-ARR*, *GIDI*, *EIN3*, *TCH4*, *JAZ*, and *NPR1*, exhibited more pronounced upregulation in HX compared to HS when cold stress persisted for more than 7 days (**Figure 6B**).

#### 3.3.5 Starch and sucrose metabolism pathways analyses

The KEGG enrichment analysis indicates that many of the DEGs between the two cultivars under different cold treatments are associated with starch and sucrose metabolism. **Figure 7A** shows a schematic diagram of the metabolic pathways for starch and sucrose. In all, 39 DEGs were selected for this study. These DEGs encode enzymes such as beta-fructofuranosidase (INV), hexokinase (HXK), fructokinases (FRK), glucan endo-1,7-beta-D-glucosidase (EGLC), beta-glucosidase (Bglu), sucrose-phosphate synthase (SPS), UTP-glucose-1-phosphate uridylyltranferase, (UGP2), ADP-sugar diphosphatase (NUDX14), trehalose 1–4-phosphate synthase (otsA), glycogen phosporylase (PYG), trehalose 11-phosphate phosphatase (otsB) beta-amylase (BAMY), and endoglucanase (EG). Among these genes, one *INV* gene, one *EGLC* gene, and one *NUDX14* gene exhibited significantly lower expression levels in LT-HX compared to LT-HS. However, the expression of most DEGs was significantly higher in LT-HX than in LT-HS, particularly in the 25-day treatment (**Figure 7B**). The higher expression levels may lead to better cold tolerance in HX.

### 3.4 Combined transcriptomic and metabolomic analyses

We combined our metabolomic and transcriptomic data to further clarify the responses of HS and HX to cold stress. Common pathways established based on the KEGG pathways of DEGs and DEMs include the flavone and flavonol biosynthesis, flavonoid biosynthesis, pyrimidine metabolism, and purine metabolism pathways. Of these, the phenylpropanoid biosynthesis pathway plays an important role in regulating cold stress. This pathway was significantly enriched in both the metabolomic and transcriptomic data, with six significantly different metabolites and eight key enzymes encoded by 24 DEGs. While most metabolites, including phenylalanine, p-coumaryl alcohol, ferulic acid, coniferyl alcohol, coniferin, sinapyl alcohol, caffeic acid, and succinate, were upregulated in both cultivars under cold stress, p-coumaroyl quinic acid consistently declined. Moreover, the expression of some metabolites, such as p-coumaryl alcohol, coniferyl alcohol, coniferin, sinapyl alcohol, and succinate, was significantly higher in HX than in HS after 25 days of cold treatment (**Figure 8A**). Most DEGs exhibited different expression patterns in the two cultivars. Under cold stress, two genes encoding 4-coumarate–CoA ligase (4CL), four genes encoding shikimate O-hydroxycinnamoyltransferase (HCT), one gene encoding cinnamoyl-CoA reductase (CCR), three genes encoding cinnamyl-alcohol dehydrogenase (CAD), and five genes encoding coniferyl-aldehyde dehydrogenase (CALDH) exhibited higher transcription levels in HS than in HX. However, the expression of some genes was higher in the LT-HX group than in the LT-HS group, including two genes encoding phenylalanine ammonia-lyase (PAL), which is the entry enzyme for phenylpropanoids, one gene encoding caffeic acid 3-O-methyltransferase/acetylserotonin O-methyltransferase (COMT), two genes encoding ferulate-5-hydroxylase (F5H), and two genes encoding CAD (**Figure 8B**). The results of this analysis suggest that these upregulated DEMs and DEGs, which are related to phenylpropanoid biosynthesis, may improve cold tolerance in *C*. *oleifera*.

## 4 Discussion

Temperature affects plant growth and development and limits species’ geographical distributions, particularly during the critical stages of reproduction. To survive in extreme environments, plants have evolved adaptive responses whereby they regulate developmental processes, such as flowering or dormancy, in response to variation in temperature (Susila et al., 2018). Previous studies have demonstrated that the cultivation of *Camellia sinensis* has been constrained by extreme temperatures, and that cold stress might cause flowers and fruits to drop, leading to reduced yields (Ru and Ju, 2010). The risk for cold damage can be reduced, and the production of *C*. *oleifera* increased, by developing new varieties. In this study, we sought to unravel the different regulatory mechanisms involved in cold stress responses in HS and HX through a combination of physiological, transcriptomic, and metabolomic analyses. The results indicate that, in addition to physiological changes, numerous DEGs and DEMs were present in the flower buds of the two cultivars after the cold treatments. We found that, in contrast to the trend for auxin, the contents of D-fructose, glucose, sucrose, and ABA in the flower buds of HX were significantly higher than in HS at after 25 days of cold treatment. Furthermore, KEGG enrichment analysis indicated that some DEGs are related to the starch and sucrose metabolism, phenylpropanoid biosynthesis, and plant hormone signal transduction pathways; most of these DEGs exhibited higher expression in HX than in HS after 25 days of cold stress. These results are consistent with our phenotypic observations: HX bloomed first and produced a sticky, nectar-like material at the base of the flowers and columns. By contrast, some flower buds in HS entered dormancy, whereas others remained closed, and flowering was delayed.

### 4.1 Transcriptional regulation-related genes

Stress resistance in plants is regulated by multiple genes. Among these, TFs play the important role of a molecular switch in the transcriptional regulation network of stress responses, mainly regulating the function of downstream genes *via* interactions with cis acting elements in downstream target gene promoters (Liu et al., 2013). Several TFs, including MYB, WRKY, NAC, and AP2, are associated with cold tolerance in plants (Khan et al., 2018). Of these, MYBs play key roles in regulating responses to a variety of abiotic stresses (e.g., low temperatures, drought, and high salt). For example, in a previous study, overexpression of the gene *OsMYB4* in *Arabidopsis thaliana* led to markedly enhanced cold tolerance (Vannini et al., 2004). By contrast, overexpression of *VcMYB4a* in blueberry led to increased cold sensitivity, suggesting that MYB genes may negatively regulate cold tolerance through different signaling pathways (Zhang et al., 2020). *ZmWRKY106* in maize (*Zea mays*) is induced by drought stress, and its promoter region contains C-repeat/dehydration response element (DRE), low-temperature response element (LTR), and other important stress response elements. Both high-temperature stress and exogenous ABA can significantly induce the expression of *ZmWRKY106* through the ABA signal pathway (Wang et al., 2018). In addition, the AP2/ERF genes, such as *BpERF13*, are also related to cold stress; overexpressed (OE) transgenic lines of *Betula platyphylla* upregulate CBF genes and mitigate reactive oxygen species (ROS) under cold treatment (Lv et al., 2020). *CaNAC064*, in the NAC family, is a crucial regulator of cold stress tolerance in peppers, whereas *PbeNAC1* plays an important role in improving the cold tolerance of *Pyrus betulifolia* in cold environments (Jin et al., 2017; Hou et al., 2020). Our transcriptomic analysis revealed that the majority of DEGs between HX and HS during the later stages of cold stress belong to the MYB, WRKY, NAC, and AP2 families, confirming that cold resistance in *C*. *oleifera* is regulated by multiple TF genes, and that genes from these four families play key roles in cold resistance. Further research is required to understand the cold resistance mechanisms of TFs in *C*. *oleifera*.

### 4.2 Plant hormone signal transduction pathways involved in cold resistance

Among the complex mechanisms by which plants adapt to cold stress, the hormone system is critical (Zhao et al., 2021). Growth regulators include auxins, GA, cytokinin, ABA, ET, SA, JA, and BR. Of these, ABA and auxin play a key role in the signal transduction processes involved in plant resistance to abiotic stress (Eremina et al., 2016). ABA plays an important role in mediating cold perception and promoting cold tolerance in *Populus euphratica* (Chen et al., 2014) Furthermore, exogenous ABA can induce various cold tolerance mechanisms in the cells and seedlings of cold-sensitive rice (Shinkawa et al., 2013). Our hormone content measurements indicate that ABA is a significant component of the cold stress responses of *C*. *oleifera*. ABA is essential for plant growth and development, and response mechanisms to cold stress may be either ABA-dependent or ABA-independent (Tuteja, 2007). The ABA receptors *PP2C* and *SnRK2* are important regulators that can play either positive or negative regulatory roles in different plant species (Yang et al., 2017). Both *PP2C* and *SnRK2* were differentially expressed in *C*. *oleifera*, indicating that the species’ cold signal transduction pathway may be ABA-dependent. Moreover, the expression levels of *PYR/PYL*, *PP2C*, and *SnRK2* and the content of ABA were higher in HX than in HS after 25 days of cold stress, indicating that the ABA signal transduction pathway plays a larger role in cold signal transmission in HX.

Recent studies have found that auxin is heavily involved in the cold stress responses of plants. For example, a large number of auxin-regulated genes in *Brassica napus* are induced by cold stress (Guan et al., 2019). Genes encoding Aux1, GH3, and SaUR are significantly upregulated in both *Capsicum pubescens* (a cold-tolerant pepper) and *C*. *chinense* (a cold-sensitive pepper) after 12 h of cold treatment; however, the expression of these genes is significantly higher in *C*. *pubescens* (Gao et al., 2022). Similarly, the DEGs encoding AUX/IAAs are both up- and downregulated at 4°C in a cold-tolerant rice cultivar (Zhao et al., 2015). In our analyses, two AUX/IAA genes were downregulated during the long-term cold treatment, and five were upregulated. These results corroborate those of other studies, and imply that auxin-related genes are associated with cold tolerance in *C*. *oleifera*. Furthermore, most genes exhibited higher expression in HX than in HS after 25 days of cold stress. Conversely, context of auxins such as IAA exhibited contrasting trends in the two cultivars, suggesting that auxin-responsive genes may negatively regulate the synthesis of auxin, and that auxin hormone signal transduction is more active in HX than HS.

### 4.3 Starch and sucrose metabolism in response to cold stress

Carbohydrate metabolism is critical to plant growth and tolerance of environmental stress (Sharma et al., 2021). Remobilization of starch and sucrose releases energy, sugars, and derived metabolites, which helps to alleviate abiotic stress, and is a fundamental process in plant adaptation (Thalmann and Santelia, 2017). In this process, key enzyme genes involved in starch and sucrose metabolism, including β-glucosidase genes (*Bglu*s), β-mylase genes (*BAMY*s), hexokinase genes (*HXK*s), sucrose phosphate synthetase genes (*SPS*s), and sucrose invertase genes (*INV*s), contribute to cold stress responses by regulating osmotic adjustment ability *via* the accumulation of soluble sugars, or by inducing the expression of cold resistance genes and key enzyme genes associated with the antioxidant system (Cao et al., 2014). For example, the beta-glucosidase gene in kiwi fruit is induced by cold stress and exhibits significantly different expression patterns under cold treatments. Similarly, the transcription levels of the glucosidase gene in chickpea (*Cicer arietinum*) increase considerably under cold stress, thus conferring significantly increased resistance (Khazaei et al., 2015; Sun et al., 2021). Furthermore, *BAMY* genes play a key role in plant responses to cold stress by degrading starch and regulating the accumulation of soluble sugars (Ma et al., 2022). For example, overexpression of *PbrBAM3* in tobacco (*Nicotiana tabacum*) and pear (*Pyrus ussuriensis* and *P*. *betulifolia*) increase *BAM* activity and thereby have a positive effect on cold tolerance (Zhao et al., 2019; Liang et al., 2021). *HXK*-related genes play important roles in sugar sensing and signal transduction. *HXK*s are more significantly induced in a cold-tolerant cultivar of *Jatropha curcas* (SCZ) than in the cold-sensitive cultivar YH9. In addition, overexpression of *SPS* and *INV* contribute to cold tolerance by influencing sugar accumulation (Bhowmik et al., 2006; Dahro et al., 2016).

In our analyses, several key enzyme genes related to starch and sucrose metabolism, including *INV*s, *HXK*s, *FRK*s, *SPS*s, *Bglu*s, and *BAMY*s, were involved in cold responses. Most of these genes were induced in HX, and their expression was higher in HX than in HS after 25 days of cold stress, which is consistent with changes observed in the contents of sucrose, D-fructose, and glucose. These results indicate that differences in the cold responses of HS and HX may be attributable to differences in sugar accumulation that are regulated by multiple genes. Moreover, the accumulation of sugars in the flower buds was more pronounced in HX than in HS after 25 days of treatment, potentially providing energy to the flowers of HX and preventing cold stress from affecting flowering. This may explain why the buds of HX opened normally whereas those of HS opened more slowly or not at all, and indicates that HX flower buds are less affected by cold stress. This finding may contribute to improving cold tolerance in *C*. *oleifera*.

### 4.4 Phenylpropanoid biosynthesis involved in cold resistance

Our integrated transcriptomic and metabolomic analysis revealed significant enrichment of DEGs and DEMs in the phenylpropanoid biosynthesis pathway. Phenylpropanoid metabolism is among the most important metabolic pathways in plants, and the metabolites from this pathway affect development and plant–environment interactions (Dong and Lin, 2021). Cold stress causes excess accumulation of ROS in the cell membrane, leading to cell damage. Phenolic compounds use electrons and hydrogen atoms to flush ROS and prevent lipid peroxidation (Rezaie et al., 2020; Devireddy et al., 2021). Phenolic biosynthesis responds to cold stress by enhancing the expression of key genes encoding phenylalanine ammonia-lyase (PAL), cinnamyl-alcohol dehydrogenase (CAD), and hydroxycinnamate transferase (HCT) (Sharma et al., 2019). Integrated transcriptomic and metabolomic analyses have demonstrated that genes related to PAL and coniferin exhibit higher expression in the cold-tolerant peanut (*Arachis hypogaea*) cultivar SLH than in the cold-sensitive cultivar ZH12 (Wang et al., 2021). In addition, the expression of *PAL* genes is higher in a cold-tolerant variety of Tartary buckwheat (TM) than in the sensitive variety RG (Raza et al., 2021). Sinapyl alcohol is among the precursors of lignin, which is associated with water and solute transport and cell wall rigidity, and contributes to abiotic stress resistance (Lee et al., 2021). *Hosta ventricosa* enhances its cold resistance by adjusting the ratio of sinapyl alcohol to coniferyl alcohol, thereby altering the morphological structure of the cell wall (Zhuang et al., 2021). Furthermore, sinapyl alcohol is significantly upregulated in the tolerant *Zea mays* cultivar B144 (Yu et al., 2022). In addition, succinate accumulates when plants are exposed to cold stress (Song et al., 2016; Xie et al., 2022). We found that DEMs, including coniferin, coniferyl alcohol, sinapyl alcohol, and succinate, along with related genes, exhibited similar positive trends under cold stress, and that these genes were more strongly expressed in HX than in HS after 25 days of cold treatment. The results indicate that the aforementioned genes and metabolites are associated with improved cold tolerance in the flowers buds of *C*. *oleifera*. Moreover, transcriptional regulation and metabolism were more active in HX flower buds under long-term cold stress, which might explain why HX flower buds opened normally whereas most HS flower buds delayed opening or did not open at all. This suggests that the flower buds of HX are less affected by cold stress than those of HS. While this contradicts the results of a previous study (Wu et al., 2020a), the contradiction is likely attributable to differences in test materials: that study used *C*. *oleifera* leaves, whereas we used flower buds, and vegetative organs (leaves) and reproductive organs (flower buds) differ in their responses to cold stress. Therefore, cold resistance in different *C*. *oleifera* cultivars may vary among tissues and organs.

## 5 Conclusion

Cold stress negatively impacts plant growth and development and leads to changes at the phenotypic, physiological, and molecular levels. In this study, differences in response mechanisms in the flower buds of two *C*. *oleifera* cultivars (HS and HX) under cold stress were investigated from physiological, transcriptomic, and metabonomic perspectives. Metabolites, including coniferin, coniferyl alcohol, succinate, and sinapyl alcohol, accumulated significantly in response to the cold treatments, and expression of related genes increased. Effects were more pronounced in HX than in HS during the later stages of the treatments, which may explain why the flower buds of HX opened normally whereas most HS flower buds either delayed opening or failed to open at all. Our results indicate that HX maintains higher levels of carbohydrate metabolism in the flower buds than does HS, and that the flower buds of HX are less strongly affected by cold stress. The cold resistance of *C*. *oleifera* cultivars varies among tissues and organs. The genes and metabolic processes identified in this study provide valuable information for future molecular breeding.

## Data availability statement

The data presented in the study are deposited in the Figshare repository, and the link is 10.6084/m9.figshare.21775748.

## Author contributions

Y-JW and L-LW have contributed equally to this work and share first authorship. Y-JW analyzed the data as well as and writing the original draft, L-LW designed and performed the experiments, writing review and revising, as well as funding acquisition. M-HS and ZL writing review and edited the manuscript and J-AL and X-FT revised and proofread the paper. All authors contributed to the article and approved the submitted version.

## Supplementary material

Supplementary Table 1 Absolute quantification of 13 sugars and three plant hormones detected in HS and HX at different times of cold stress.

Supplementary Table 2 DEGs detected in HS and HX at different times of cold stress.
