# Supplementary material for: Transcriptomic and metabolomic insights on the molecular mechanisms of flower buds in responses to cold stress in two Camellia oleifera cultivars
Source: Front Plant Sci. 2023 Feb 20;14:1126660. doi: 10.3389/fpls.2023.1126660 (PMC10037702; doi:10.3389/fpls.2023.1126660)
Supplement: Supplementary Table 2 — DEGs detected in HS and HX at different times of cold stress. [file DataSheet_2.docx]

***Supplementary Material***

**1. Supplementary Figures**

**
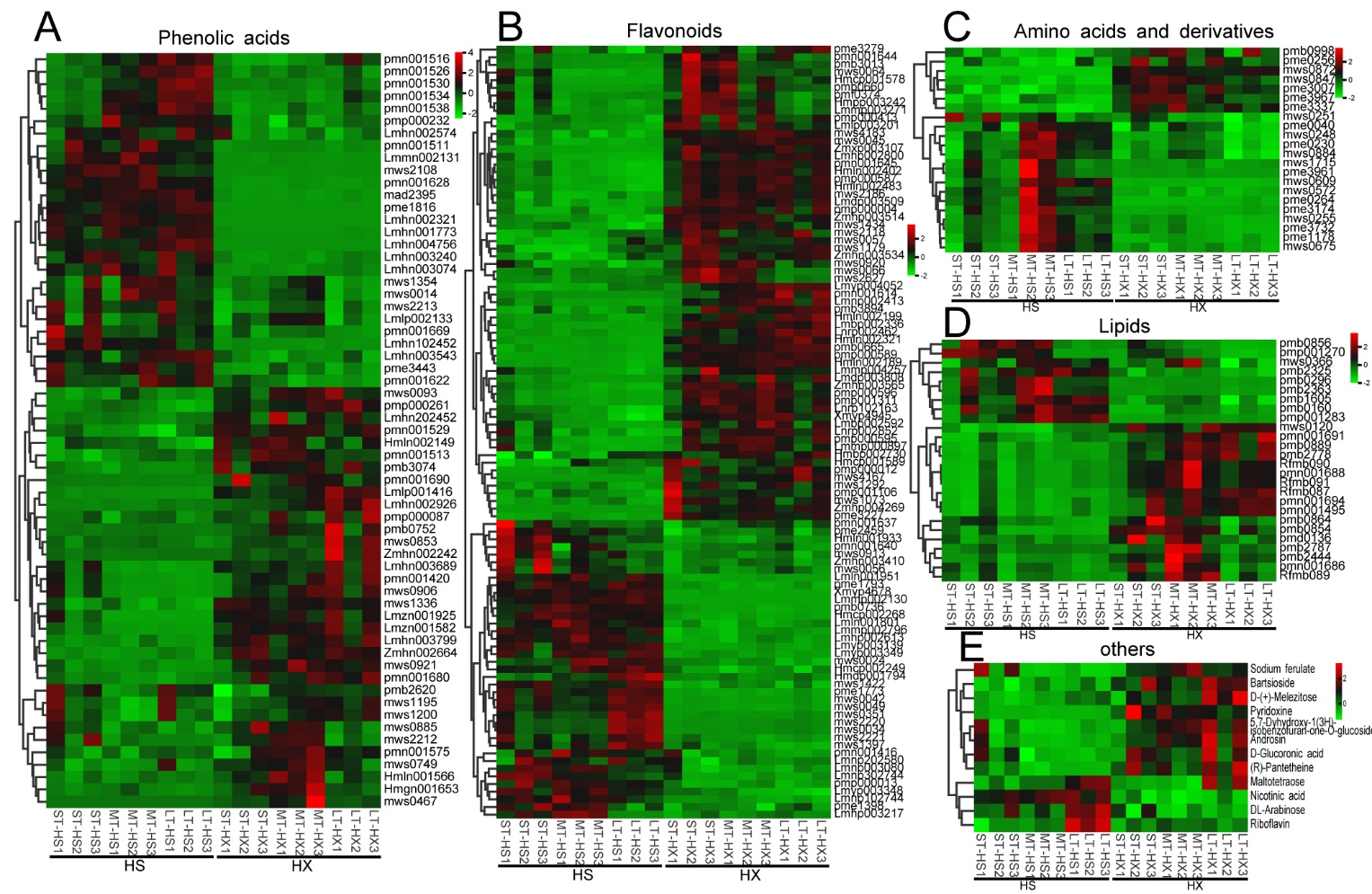
**

**Supplementary Figure 1**: Heat map of metabolites. phenolic acids(A); Flavonoids(B); amino acids and derivatives(C); lipids(D); others(E) content responsive to cold stress in HS and HX.


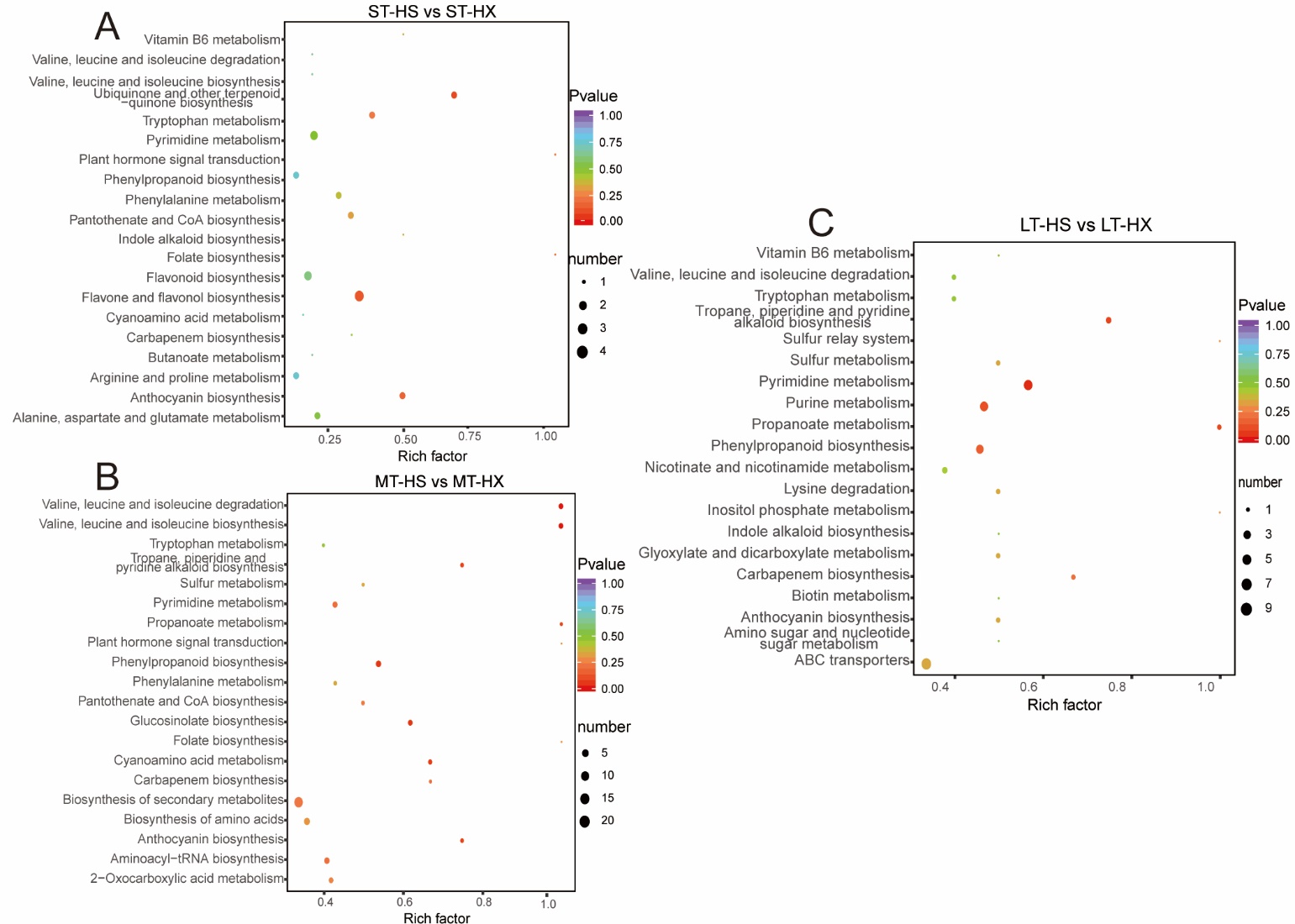


**Supplementary Figure 2：**KEGG enrichment analysis of DEMs after (A) 1, (B) 7, and (C) 25 days of cold stress treatments.


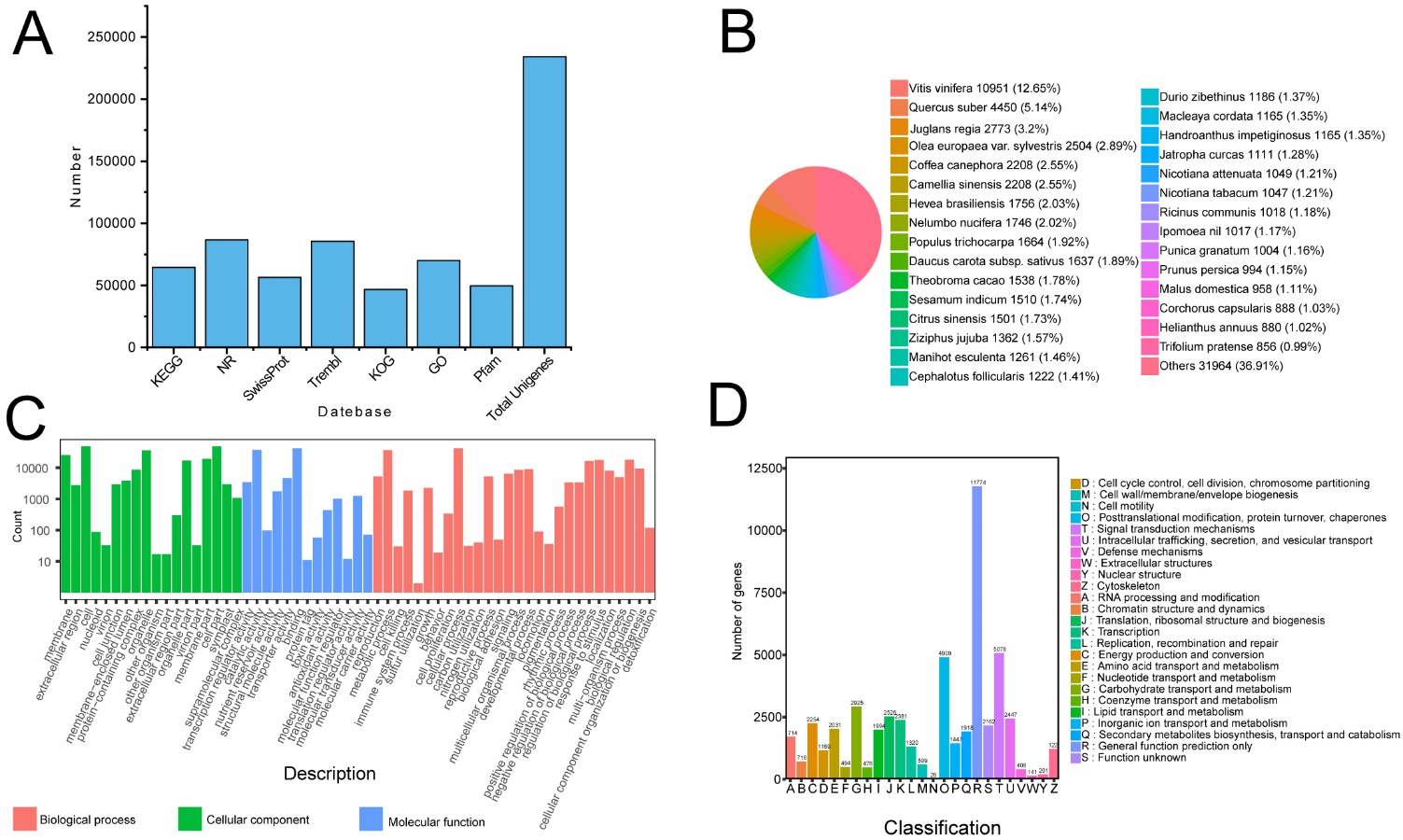


**Supplementary Figure 3：**Analysis of the transcriptome data. (A) Unigenes annotated statistics chart. (B) Pie chart presenting unigenes annotation in NR database. (C) Bar chart showing unigenes annotation in GO database. (D) Bar chart showing the classification of unigenes in KOG database.
